# Phloem-specific translational regulation of soybean nodulation: Insights from a phloem-targeted TRAP-seq approach

**DOI:** 10.1093/plphys/kiaf570

**Published:** 2025-11-15

**Authors:** Jae hyo Song, Shin-ichiro Agake, Sayuri Tanabata, Yaya Cui, Li Su, Bruna Montes-Luz, Dong Xu, Gary Stacey

**Affiliations:** C.S. Bond Life Science Center, Division of Plant Sciences and Technology, University of Missouri, Columbia, MO 65201, USA; C.S. Bond Life Science Center, Division of Plant Sciences and Technology, University of Missouri, Columbia, MO 65201, USA; Institute of Global Innovation Research, Tokyo University of Agriculture and Technology, 3-8-1 Harumicho, Fuchu, Tokyo 183-8538, Japan; C.S. Bond Life Science Center, Division of Plant Sciences and Technology, University of Missouri, Columbia, MO 65201, USA; Center for International Field Agriculture, Research and Education, College of Agriculture, Ibaraki University, Ami 4668-1, Ami, Inashiki 300-0331, Japan; C.S. Bond Life Science Center, Division of Plant Sciences and Technology, University of Missouri, Columbia, MO 65201, USA; Institute for Data Science and Informatics, C.S. Bond Life Science Center, Department of Electrical Engineering and Computer Science, University of Missouri, Columbia, MO 65201, USA; C.S. Bond Life Science Center, Division of Plant Sciences and Technology, University of Missouri, Columbia, MO 65201, USA; Institute for Data Science and Informatics, C.S. Bond Life Science Center, Department of Electrical Engineering and Computer Science, University of Missouri, Columbia, MO 65201, USA; C.S. Bond Life Science Center, Division of Plant Sciences and Technology, University of Missouri, Columbia, MO 65201, USA

## Abstract

Soybean (*Glycine max*) root nodulation is a symbiotic process that requires complex molecular and cellular coordination. The phloem plays a crucial role not only in nutrient transport but also in long-distance signaling that regulates nodulation. However, the molecular mechanisms underlying phloem-specific regulation during nodulation remain poorly characterized. Here, we developed a phloem-specific Translating Ribosome Affinity Purification sequencing system to investigate the translational dynamics of phloem-associated genes during nodulation. Using a phloem-specific promoter (*Glyma.01G040700*) combined with the GAL4-UAS amplification system, we successfully captured the translatome of soybean root phloem at early (72 h postinoculation, hpi) and late (21 d postinoculation, dpi) nodulation stages. Differential expression analysis revealed dynamic translational reprogramming, with 2,636 differentially expressed genes (DEGs) at 72 hpi and 8,422 DEGs at 21 dpi. Gene ontology and pathway enrichment analyses showed stage-specific regulatory shifts, including early activation of ethylene and defense pathways and late-stage enhancement of nutrient transport and vascular development. Transcription factor analysis identified *GmbHLH121* as a key phloem-specific regulator of nodulation. Functional validation using RNA interference knockdown and overexpression experiments demonstrated that *GmbHLH121* negatively regulates nodule formation, likely acting downstream of or independently from early nodulation signaling pathways. Additionally, we uncovered dynamic regulation of cell wall-modifying enzymes (pectin methylesterase [PME] and PME inhibitors) in the phloem, implicating their role in modulating plasmodesmata permeability and facilitating symplastic connectivity during nodulation. Our findings highlight the critical role of phloem-mediated translational regulation in coordinating root nodulation, emphasizing the phloem as an active regulatory hub for long-distance signaling and symbiotic efficiency.

## Teaser

Soybean phloem actively regulates root nodulation through stage-specific translational control of signaling, transport, and cell wall modification genes to coordinate symbiotic development.

## Introduction

Soybean (*Glycine max*) phloem plays a pivotal role in facilitating symbiotic interactions essential for root nodulation and nitrogen fixation. Beyond its primary function of transporting nutrients and metabolites, the phloem serves as a conduit for hormonal and signaling molecules that regulate plant development and symbiotic efficiency. For instance, autoregulation of nodulation (AON) relies on the movement of root-derived CLAVATA3/Embryo Surrounding Region-Related peptides via the phloem to the shoot, where they modulate *miR2111* expression to fine-tune nodule formation (Zhang et al. 2021). Additionally, shoot-derived cytokinins systemically regulate root nodulation in AON (Sasaki et al. 2014).

The phloem also mediates the translocation of critical secondary metabolites such as terpenoids, amino acids, and sucrose, which contribute to root growth and nodule maintenance under nutrient-limited conditions (Parsons et al. 1993; Ali et al. 2021). Recent 3D imaging studies highlight the complexity of the vascular network within nodules, reinforcing the importance of phloem in maintaining efficient nutrient and signaling molecule exchange (Livingston et al. 2019). Despite the phloem's critical role, the molecular basis of its regulatory function in nodulation remains inadequately characterized. While Translating Ribosome Affinity Purification sequencing (TRAP-seq) has been instrumental in elucidating tissue-specific translational mechanisms, prior research has focused on cortical tissues (Song et al. 2022). The translational landscape of the phloem during nodulation remains unexplored. To address this knowledge gap, we employed a phloem-specific TRAP-seq approach to delineate the regulatory pathways governing nodulation in soybean.

Legume nodulation is a highly coordinated process involving intricate molecular and cellular interactions between host plants and nitrogen-fixing bacteria. The establishment of symplastic continuity between sieve elements and developing nodule primordia is crucial for the translocation of macromolecules, including signaling molecules and transcription factors (TFs). Previous research indicated that during the early stages of nodule development in *Medicago truncatula*, the initiation of nodule primordia induces symplastic continuity between the root phloem and the nodule, facilitating the movement of essential signaling molecules (Complainville et al. 2003). Additionally, the role of callose in regulating symplastic communication has been highlighted, as it coordinates the development of symbiotic root nodules by establishing connections between nodule primordia initials (Gaudioso-Pedraza et al. 2018). Symplastic continuity is essential for intercellular communication during root nodule development, enabling signal exchange through plasmodesmata (PD). These channels are regulated by callose deposition and cell wall biomechanics. Pectin methylesterases (PMEs) contribute to PD modulation by de-methylesterifying pectin, releasing protons and methanol—methanol being a major metabolic product of PME activity (Fall and Benson 1996). PME-derived methanol promotes PD dilation and activates methanol-responsive genes such as β-1,3-glucanases, which degrade callose and facilitate viral spread (Dorokhov et al. 2012; Zavaliev et al. 2013). PME inhibitors (PMEIs) control PME activity by forming 1:1 inhibitory complexes in the apoplast. Overexpression of PMEIs reduces PME activity, increases pectin methylesterification, and limits methanol emission, thereby restricting callose degradation and PD expansion. PMEI expression is also induced by viral infection and methanol exposure, indicating a feedback mechanism to protect against pathogen movement (Kogovšek et al. 2010; Dorokhov et al. 2012; Yadav and Chattopadhyay 2014). These findings position the PME–PMEI module as a key regulator of PD permeability and symplastic signaling during both development and stress responses.

TFs expressed in the phloem orchestrate long-distance signaling by regulating hormone homeostasis and nutrient transport. Their role in coordinating rhizobial infection and nodule formation with whole-plant physiological requirements is crucial. Several families of TFs, including *Ethylene Response Factor* (*ERF*), *MYB*, *NAC*, *basic Helix-Loop-Helix* (*bHLH*), and *Auxin Response Factor* (*ARF*), have been implicated in various stages of nodulation, from rhizobial infection to mature nodule function. Early studies demonstrated that *ERF* TFs are integral to nodulation by mediating ethylene signaling, a key regulator of nodule number and function. Specifically, *ERN1* (*ETHYLENE RESPONSE FACTOR REQUIRED FOR NODULATION 1*) was shown to be a key positive regulator of Nod factor signaling in legumes (Middleton et al. 2007; Kawaharada et al. 2017). Similarly, *MYB* TFs have been linked to nodulation through their involvement in root hair infection and meristem formation, as seen in *LjMYB14*, which regulates flavonoid biosynthesis essential for rhizobial interaction in *Lotus japonicus* (Shelton et al. 2012). More recent research demonstrated that *GmMYB176* regulates isoflavonoid synthesis in soybean nodulation, emphasizing the conservation of *MYB* function in different legumes (Anguraj Vadivel et al. 2021). In addition, *MYB36*-dependent Casparian strip (CS) formation in *L. japonicus* was shown to be essential for effective root-to-shoot CEP1 signaling and successful nodule development and nitrogen fixation, highlighting a broader regulatory role of TFs in nodulation (Shen et al. 2025). *NAC* TFs have also emerged as key players in the nodulation process, primarily through their role in stress responses and symbiotic regulation. For instance, *MtNAC969* was shown to regulate nodule senescence by modulating stress responses in *M. truncatula* (De Zélicourt et al. 2012). *GmNAC181* promotes symbiotic nodulation and salt stress tolerance of nodulation in soybean by directly activating *GmNINa* expression, underscoring its central role in coordinating environmental and developmental signals during nodulation (Wang et al. 2022b). Additionally, other NAC TFs such as *GmNAC039* and *GmNAC018* have also been implicated in regulating nodule development, suggesting a broader role of NAC TFs in the control of legume-rhizobia symbiosis (Yu et al. 2023).

*ARF* TFs, which mediate auxin-responsive gene expression, have been implicated in the regulation of nodulation. For instance, recent studies demonstrated that expression of *GmARF8a* and *GmARF8b* is tightly controlled by *miR167c*, and this expression is essential for optimal nodule formation (Wang et al. 2015). The *bHLH* family has been implicated in root architecture modification, influencing lateral root development and nodule positioning. Studies on *MtbHLH1* indicate its role in regulating root hair growth and rhizobial attachment (Godiard et al. 2011). Furthermore, *GmbHLH300* was demonstrated to regulate iron homeostasis in soybean nodules, indicating an additional layer of metabolic regulation by *bHLH* TFs (Wu et al. 2023). While the roles of these TFs in nodulation are well known, their specific contributions within the phloem in coordinating systemic and localized nodule development remain unclear. Our study reveals dynamic changes in the expression of *ERF*, *MYB*, *NAC*, *bHLH*, and *ARF* TFs in the phloem during early and late nodulation stages, suggesting that phloem-mediated regulation plays a crucial role in integrating long-distance signaling with nodule formation. Collectively, our findings emphasize the importance of phloem-mediated transcriptional regulation in optimizing nodule development, linking systemic regulatory networks with root symbiosis.

## Results

### Development and validation of a phloem-specific TRAP-seq system

**Figure 1.**
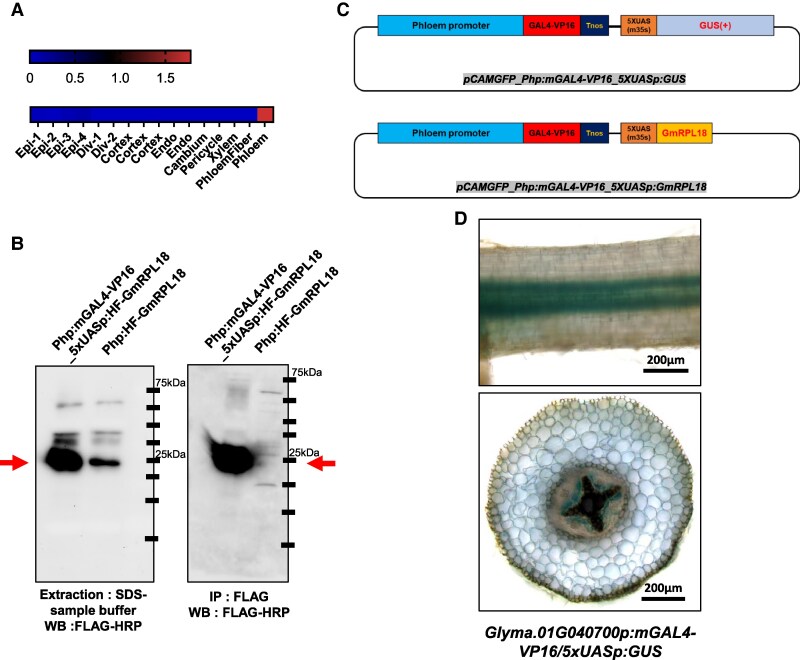
Phloem-specific TRAP-seq system design and experimental validation. **A)** In silico scRNA-seq data illustrating phloem-specific expression of *Glyma.01G040700*, confirming promoter suitability. **B)** Western blot analysis confirming HF-GmRPL18 protein expression (left) and successful immunoprecipitation of ribosome complexes (right), validating the functionality of the TRAP system in phloem tissue. **C)** Schematic representation of TRAP-seq constructs. A phloem-specific promoter (Php) drives the expression of GAL4-VP16, which activates UAS-controlled GUS or HF-GmRPL18 for promoter validation and ribosome tagging, respectively. **D)** GUS reporter assay validating promoter activity. Strong phloem-specific GUS signals are observed in root longitudinal sections (top) and transverse sections (bottom). Scale bar, 200 *µ*m.

The TRAP-seq technique enables precise analysis of tissue-specific translatomes by leveraging a His-FLAG-tagged ribosomal protein L18 (HF-GmRPL18). This system facilitates the immunoprecipitation of actively translating polysomes and their associated mRNAs, providing a robust method for studying translation at a tissue-specific level (Zanetti et al. 2005). To establish a phloem-specific TRAP-seq system, we first identified a suitable phloem-specific promoter using laser capture microdissection (LCM) coupled with RNA-seq. Phloem tissues were isolated from soybean roots, and analysis of this LCM RNA-seq dataset identified the *Glyma.01G040700* promoter as phloem-specific. Published single-cell RNA (scRNA)-seq data (Cervantes-Pérez et al. 2024) further validated its strong and selective expression in phloem cells, supporting its use for phloem-targeted applications (Fig. 1A). Despite its high specificity, the intrinsic activity of the *Glyma.01G040700* promoter was insufficient to achieve the expression levels necessary for efficient translatome capture (Fig. 1B). Robust promoter activity was essential to enhance the sensitivity of ribosome-associated mRNA isolation and ensure adequate transcript recovery for downstream analyses.

To overcome this limitation, we implemented the GAL4-UAS system, which significantly amplified promoter-driven expression while preserving tissue specificity (Fig. 1C). In this system, *GAL4-VP16* was placed under the control of the *Glyma.01G040700* promoter, leading to a substantial increase in HF-GmRPL18 expression, as confirmed by immunoblot analysis in transgenic roots. This optimized system provided enhanced translatome capture efficiency, enabling detailed studies of phloem-specific gene regulation. To validate system functionality, we performed GUS reporter assays, which confirmed phloem-specific promoter activity (Fig. 1D). Additionally, TRAP-seq experiments were conducted at 72 h postinoculation (hpi) and 21 d postinoculation (dpi) to analyze dynamic gene expression changes in the phloem during nodulation. Immunoblot analysis of HF-GmRPL18-expressing transgenic roots demonstrated sufficient protein accumulation for efficient immunoprecipitation, validating the system's capacity to selectively capture ribosome-associated mRNAs with high specificity and reliability (Fig. 1B).

To further assess the phloem specificity of our TRAP-seq data, we compared the resulting differentially expressed genes (DEGs) with a publicly available scRNA-seq dataset of soybean root tissues (Cervantes-Pérez et al. 2024). Under commonly applied filtering thresholds (TRAP-seq: read count ≥10; scRNA-seq: normalized expression >0.1 in ≥5% of cells), ∼96% of genes enriched in phloem (Cluster #16) and phloem fiber (Cluster #15) in the scRNA-seq dataset were also detected in our phloem TRAP-seq data (Supplementary Fig. S1). This strong concordance supports the conclusion that the transcripts captured by our TRAP-seq system predominantly originate from phloem cell types, reinforcing the tissue specificity and reliability of our approach.

### Time-dependent translational reprogramming in soybean root phloem

**Figure 2.**
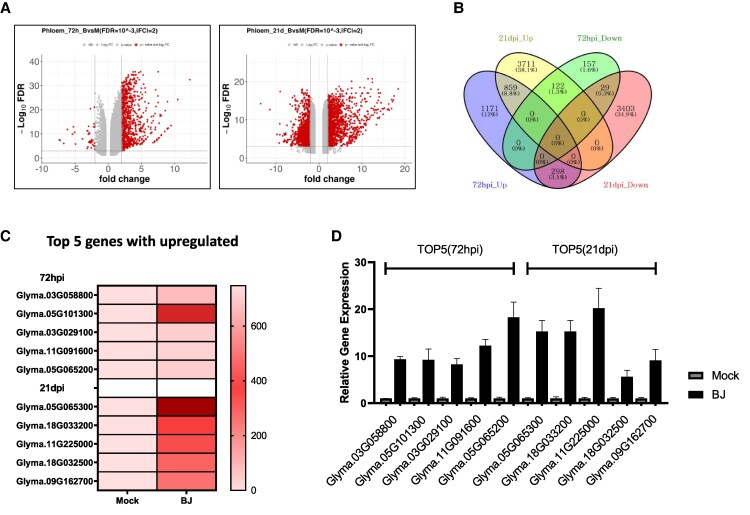
Differential gene expression analysis in soybean root phloem using TRAP-seq and validation. **A)** Volcano plots of DEGs in root phloem at 72 hpi (left) and 21 dpi (right) with mock and rhizobacterial treatment. Significant genes (FDR < 0.1, |log_2_ FC| ≥ 2) are shown in red. **B)** Venn diagram showing overlaps of upregulated and downregulated genes between 72 hpi and 21 dpi conditions. **C)** Heatmap of normalized RNA-seq expression values for the top 5 upregulated genes at 72 hpi and 21 dpi. The color scale represents the average expression levels. **D)** Bar graph showing RT-qPCR validation of the top 5 upregulated genes, displaying relative expression levels compared to mock conditions. Data are presented as mean values from 3 biological replicates (*n* = 3), with error bars indicating the standard error (Se).

This study aimed to investigate the translational dynamics in soybean root phloem during early (72 hpi) and late (21 dpi) stages of nodulation in response to rhizobial infection. The extent of translational reprogramming, visualized in the volcano plot (Fig. 2A), was determined by identifying 2,636 DEGs at 72 hpi and 8,422 DEGs at 21 dpi under the criteria of false discovery rate (FDR) < 0.01 and |log_2_-fold change| > 1 (Supplementary Table S1). A Venn diagram analysis quantified the shifts in translational activity across nodulation stages. At 72 hpi, 1,171 and 157 DEGs were uniquely upregulated and downregulated, respectively, while at 21 dpi, 3,711 and 3,403 were upregulated and downregulated. Among these, 122 DEGs transitioned from downregulated at 72 hpi to upregulated at 21 dpi. Additionally, 859 DEGs were consistently upregulated at both time points, 298 DEGs shifted from upregulated at 72 hpi to downregulated at 21 dpi, and 29 DEGs remained downregulated throughout (Fig. 2B; Supplementary Table S2). These findings highlight dynamic translational regulation in soybean root phloem during nodulation. A heatmap of normalized TRAP-seq data illustrated the extent of induction for the top 5 upregulated DEGs at each time point, demonstrating their significant expression in rhizobium-inoculated samples (Fig. 2C). RT-qPCR was subsequently performed to confirm these findings, showing strong agreement with TRAP-seq data (Fig. 2D). The consistency between RT-qPCR and TRAP-seq results reinforces the reliability of translational profiling in capturing stage-specific regulatory changes.

### Functional genomics and metabolic pathways underlying root phloem's role in nodulation

To elucidate the translational regulatory mechanisms governing phloem function during nodulation, we conducted Kyoto Encyclopedia of Genes and Genomes (KEGG) pathway and gene ontology (GO) enrichment analyses to identify key biological processes enriched in phloem-associated transcripts at 72 hpi and 21 dpi, using REVIGO (Supek et al. 2011) (REduce and Visualize Gene Ontology) to summarize and reduce redundancy in GO terms for clearer interpretation (Supplementary Tables S3 and S4).

**Figure 3.**
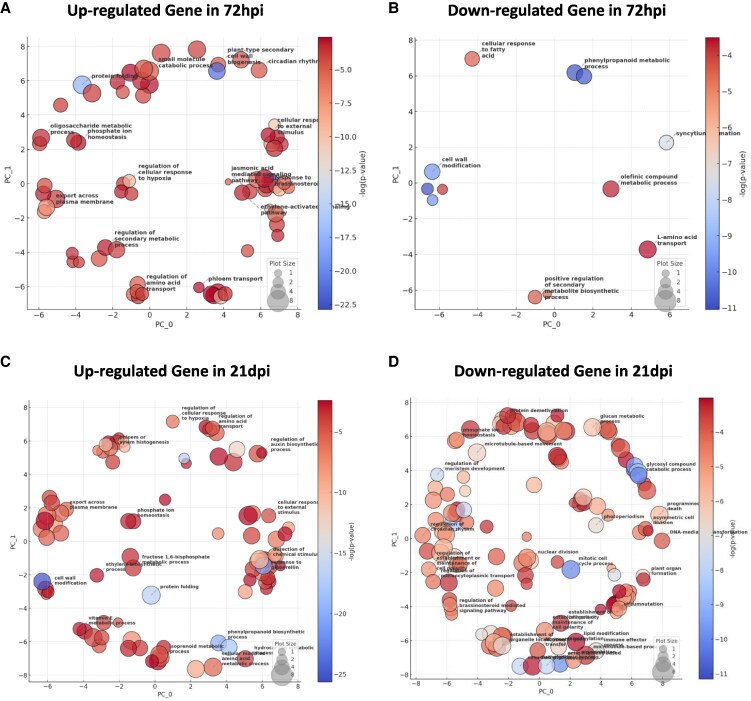
GO enrichment analysis of DEGs in soybean root phloem. REVIGO visualization of biological process GO enrichment based on DEGs at 72 hpi and 21 dpi. Each sphere represents a GO term, positioned by semantic similarity, with color indicating −log_10_(*P*-value) and size representing term frequency. **A)** Upregulated genes at 72 hpi, **B)** downregulated genes at 72 hpi, **C)** upregulated genes at 21 dpi, **D)** downregulated genes at 21 dpi.

At 72 hpi, upregulated pathways fell into 3 main categories: (i) structural and defense responses, including cell wall biogenesis and ethylene signaling, which facilitate early nodulation-related adaptations and the formation of a symplastic network; (ii) hormonal and regulatory pathways, such as jasmonic acid and brassinosteroid signaling, that coordinate nodulation responses and cell communication; and (iii) transport functions, including phloem transport and amino acid regulation, establishing initial nutrient mobilization essential for nodule development (Fig. 3A; Supplementary Fig. S2A). Conversely, downregulated pathways at this stage were mainly associated with secondary metabolism, including phenylpropanoid, flavonoid, and lipid biosynthesis, reflecting a temporary suppression of energy-intensive biosynthetic activity during early phloem adaptation and defense reprogramming (Fig. 3B; Supplementary Fig. S2B). By 21 dpi, enriched pathways shift to (i) transport-related processes, such as phosphate ion homeostasis, reflecting the phloem's role in regulating nutrient movement into developing nodules; (ii) structural modifications, including vascular differentiation and cell wall modification, reinforcing nodule vasculature for sustained transport; and (iii) regulatory mechanisms like amino acid transport and mechanical stimulus response, ensuring proper coordination of mature nodule function (Fig. 3C; Supplementary Fig. S2C). Concurrently, downregulated pathways at 21 dpi indicate a shift from early proliferation to functional specialization, categorized as (i) reduced carbohydrate metabolism, including starch and sucrose metabolism, prioritizing nitrogen assimilation; (ii) decreased cell proliferation, marked by lower nuclear division and mitotic activity, indicating meristem transition; and (iii) suppressed biochemical pathways, such as glycosyl metabolism and DNA recombination, suggesting stable genomic regulation in mature nodules (Fig. 3D; Supplementary Fig. S2D). These findings highlight the role of the phloem in first establishing and later regulating the symplastic transport network essential for effective nodule function and nitrogen fixation (Complainville et al. 2003).

### Temporal and spatial expression patterns of PME and PMEI genes in soybean root nodulation

The symplastic transport network plays a fundamental role in nodule formation and function. Previous studies showed that PMEs and PMEIs regulate PD permeability by altering cell wall methylesterification and methanol emission, thereby influencing callose turnover and intercellular communication (Fall and Benson 1996; Kogovšek et al. 2010; Dorokhov et al. 2012; Zavaliev et al. 2013; Yadav and Chattopadhyay 2014). Given the observed regulation of various cell wall-modifying enzymes by bacterial infection in the phloem, we conducted a detailed analysis of the expression dynamics of PME and PMEI to elucidate their potential involvement in PD regulation.

**Figure 4.**
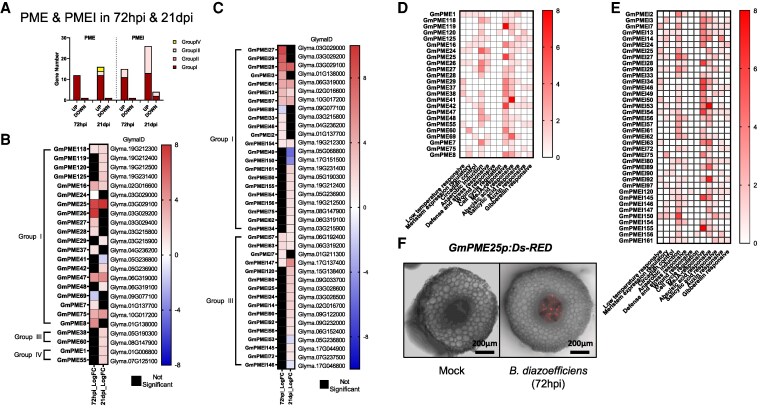
Temporal expression and regulatory analysis of PME and PMEI genes in soybean root phloem during nodulation. **A)** Classification and distribution of differentially expressed PME and PMEI genes at 72 hpi and 21 dpi. **B** and **C)** Heatmaps depicting log_2_ fold change (log_2_FC) in PME (B) and PMEI (C) gene expression over time. **D** and **E)**  *Cis*-regulatory element analysis of PME (D) and PMEI (E) promoters, with color intensity indicating element frequency. **F)** Ds-RED fluorescence assay showing *GmPME25* promoter activity in soybean root phloem at 72 hpi. Scale bar = 200 *µ*m.

Phloem TRAP-seq analysis and genome-wide studies revealed distinct temporal and spatial expression patterns of *PME* and *PMEI* genes during soybean nodulation, categorizing each gene family into 4 functional groups (Wang et al. 2020, 2021). Group I *PME* genes, significantly upregulated at 72 hpi and maintained at 21 dpi, largely overlap with *PMEI* Group I genes, both containing a PMEI domain that enables self-regulation of PME activity. This regulation balances cell wall flexibility and reinforcement, potentially contributing to PD remodeling and the establishment of symplastic connectivity during nodulation (Fig. 4, A to C; Supplementary Table S5). In contrast, several Group III and IV *PME* genes were predominantly expressed at later stages, contributing to nodule maturation and structural stabilization (Fig. 4A; Wang et al. 2021). The upregulation of both *PMEI* Group I and Group III genes at 72 hpi and 21 dpi, with most containing PME domains, suggests that self-regulation is essential for coordinated cell wall modifications (Fig. 4, A and C). These findings suggest that *PME* and *PMEI* genes not only mediate cell wall modifications but also regulate PD connectivity, ultimately influencing nodulation efficiency.

*Cis*-element analysis of *PME* and *PMEI* promoter regions using PlantCARE identified MeJA-responsive, ABA-responsive, and anaerobic induction-related elements in both *PME* and *PMEI* genes (Fig. 4, D and E; Supplementary Table S6). These findings suggest that *PME* and *PMEI* expression is modulated by jasmonic acid, abscisic acid, and oxygen availability, aligning their activity with environmental and hormonal cues during nodulation. Functional validation using *GmPME25p:Ds-RED* confirmed *GmPME25* activation in root phloem at 72 hpi, reinforcing its role in early nodule development (Fig. 4F).

**Figure 5.**
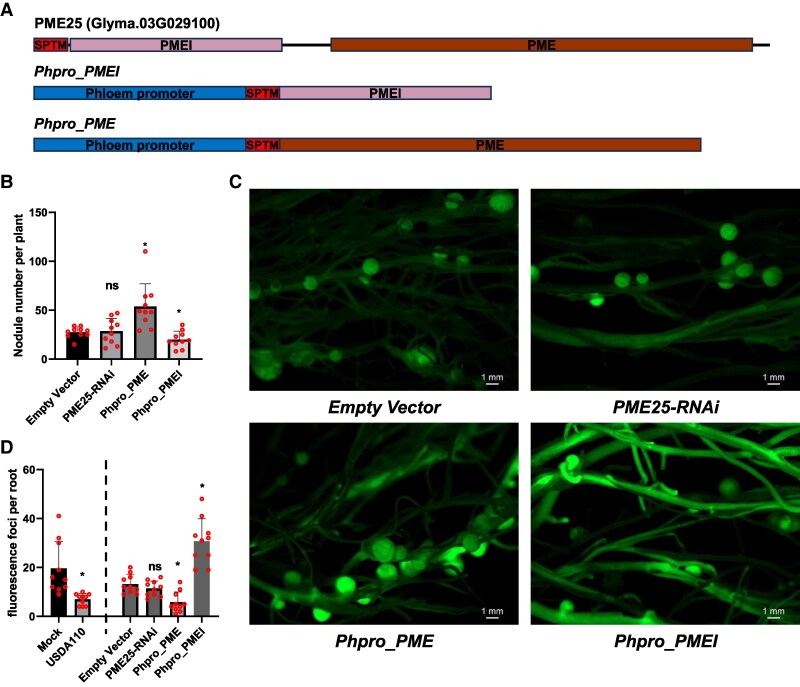
PME25 domain architecture and its effects on nodule phenotype and callose accumulation. **A)** Schematic representation of the *GmPME25* (*Glyma.03G029100*), showing the N-terminal PMEI domain (pink) and the C-terminal PME domain (brown). The signal peptide and transmembrane region (SPTM) is indicated in red. Constructs used for transgenic analysis include the PMEI or PME domain fused with the SPTM sequence and driven by the phloem-specific promoter used in the TRAP-seq system. **B)** Quantification of nodule numbers per plant in control (EV) and transgenic lines: *PME25-RNAi*, *Phpro_PMEI* (phloem-specific expression of the PMEI domain of PME25), and *Phpro_PME* (phloem-specific expression of the PME domain of PME25). Data are presented as mean values from 3 biological replicates (*n* = 3), with error bars indicating the standard error (Se). Statistical analysis was performed using 1-way ANOVA with Dunnett's test (*P* < 0.05 considered significant). **C)** Representative GFP fluorescence images showing nodule phenotypes in transgenic roots, confirming successful transformation and illustrating differences in nodule number and morphology. Scale bars = 1 mm. **D)** Quantification of callose deposition using aniline blue staining in wild-type roots (Mock, USDA110) and transgenic roots as in Supplementary Fig. S3A. Fluorescent callose foci were counted per root to assess relative callose accumulation across genotypes. Statistical analysis was performed using unpaired 2-tailed *t*-test (left) or 1-way ANOVA with Dunnett's test (right) with error bars indicate Se (*P* < 0.05 considered significant).

To further investigate the functional contribution of *PME25* to nodulation, we generated hairy roots expressing either the *PMEI* or *PME* domain of *GmPME25* under a phloem-specific promoter used for the TRAP-seq system (Fig. 5A). Phloem-specific overexpression of the *PME* domain significantly enhanced nodule number, whereas overexpression of the *PMEI* domain led to a substantial reduction (Fig. 5, B and C). Consistently, RT-qPCR analysis confirmed that PME25 transcript levels were effectively reduced in *PME25*-RNA interference (RNAi) hairy roots (Supplementary Fig. S3B). These opposing phenotypes were accompanied by corresponding changes in callose deposition, as visualized by aniline blue staining (Fig. 5D; Supplementary Fig. S3A), suggesting that modulation of PD permeability via PME/PMEI activity in the phloem influences symplastic signaling and nodulation efficiency. In contrast, *PME25-RNAi* lines did not exhibit significant changes in nodule number or callose accumulation compared with the empty vector (EV) control, possibly due to functional redundancy among PME family members.

### Dynamic regulation of transcription factors in soybean root phloem during nodulation

**Figure 6.**
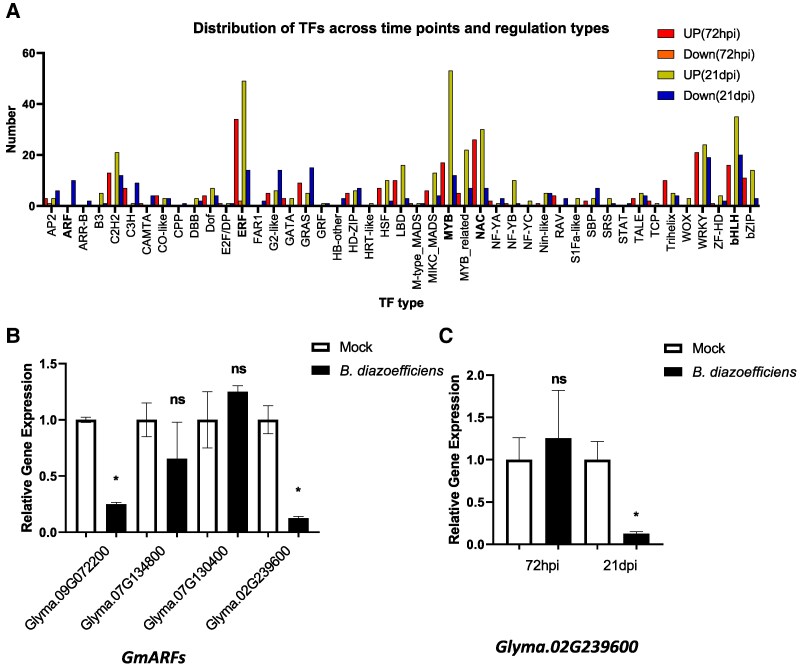
Transcriptional regulation of TFs during nodulation. **A)** Dynamic expression patterns of TFs at 72 hpi and 21 dpi, highlighting key TF families including *ARF*, *NAC*, *MYB*, *ERF*, and *bHLH*. **B)** RT-qPCR validation showing significant downregulation of *ARF* genes at 21 dpi. **C)** Time-course expression analysis of *Glyma.02G239600*, illustrating its stage-specific transcriptional regulation during nodulation. Statistical significance was evaluated by unpaired 2-tailed *t*-test; error bars indicate Se (*P* < 0.05 considered significant).

TF control plays a crucial role in the symbiosis between legumes and rhizobia. However, the temporal roles of major TFs in the phloem remain unclear despite its essential function in systemic signaling. This study analyzed TF expression in the root phloem at 72 hpi and 21 dpi, revealing stage-specific regulatory shifts (Supplementary Table S7). At 72 hpi, *ERF* (TF index number 34) exhibited the highest increase in expression, followed by upregulation of *C2H2* (TF index number: 13), *NAC* (TF index number: 26), *MYB* (TF index number: 17), *WRKY* (TF index number: 21), *bHLH* (TF index number: 16), and *LBD* (TF index number: 10) (Fig. 6A). Minimal reductions in expression were observed, with only slight suppression of *ERF* (TF index number: 2) (Fig. 6A). At 21 dpi, *ERF* (TF index number: 49) and *MYB* (TF index number: 53) showed the highest expression levels, followed by consistent upregulation of *bHLH* (TF index number: 35) and *NAC* (TF index number: 30). *C2H2* (TF index number: 21) maintained stable expression, while *ARF* (TF index number: 10) exhibited the most significant reduction. *WRKY* (TF index number: 19) and *C2H2* (TF index number: 12) were downregulated, and moderate suppression was observed in *bHLH* (TF index number: 20) and *MYB* (TF index number: 12) (Fig. 6A). In the maturation phase, *MYB* and *ERF* persist as central regulators, facilitating secondary metabolism and vascular differentiation and ensuring the developmental progression of nodules. The decline in *ARF* expression at 21 dpi suggests a regulatory mechanism that negatively regulates nodulation, whereas the reduced expression of *WRKY* and *C2H2* indicates a shift away from early-stage defense activation.

The coordinated regulation of these TFs ensures systemic signaling integration and optimized nodulation efficiency in the phloem. To comprehensively validate the stage-specific regulation of *ARF* genes, we conducted RT-qPCR analysis on 4 *ARF* genes at 21 dpi (Fig. 6B). The analysis revealed that *Glyma.09G072200* and *Glyma.02G239600* exhibited significant transcriptional repression in the 21 dpi TRAP-seq dataset, confirming their downregulation during late-stage nodulation. Furthermore, a time-course expression analysis of *Glyma.02G239600* (Fig. 6C) demonstrated a clear decline in expression, providing additional evidence of its temporal regulation. These findings suggest that ARF suppression in the phloem may be involved in regulating auxin signaling at 21 dpi, potentially contributing to nodulation.

### Identification of *GmbHLH121* as a key nodulation regulator

**Figure 7.**
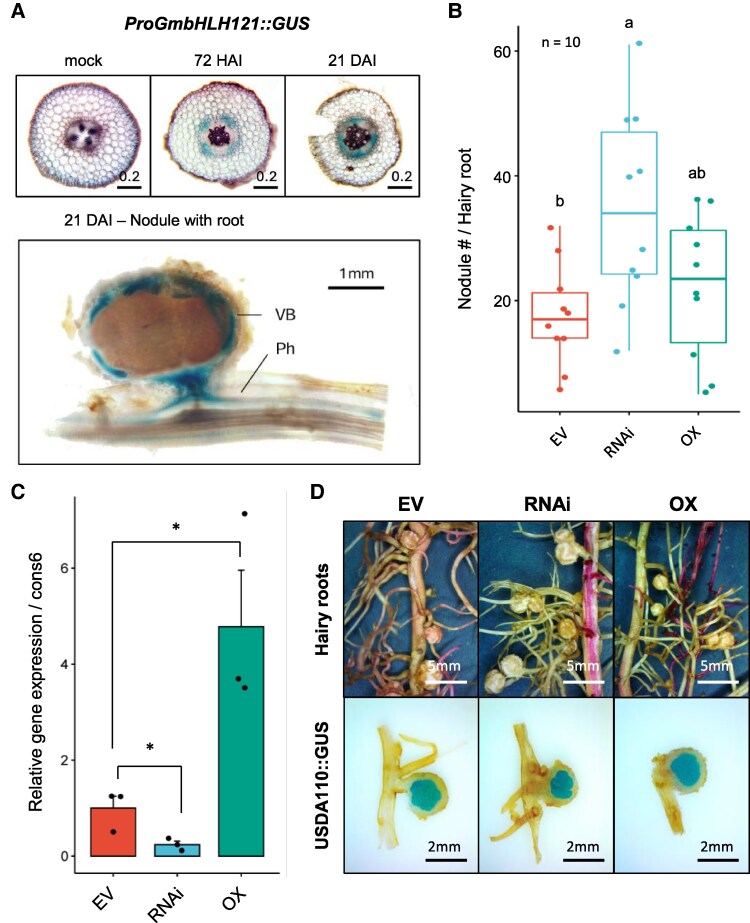
Expression and functional characterization of *GmbHLH121* in soybean nodulation. Histochemical, phenotypic, and molecular analyses of *GmbHLH121* expression in soybean roots and nodules. **A)** Cross-sections of root tissues displaying *GmbHLH121* promoter-driven GUS activity at different developmental stages, highlighting expression in vascular tissues and nodules. **B)** Nodule phenotypic analysis of *GmbHLH121* RNAi and overexpression (OX) lines, illustrating morphological differences in nodule development (*n* = 10). Boxplots show the median (center line), interquartile range (box), whiskers extending to 1.5× IQR, and outliers (points). **C)** Bar graph depicting relative *GmbHLH121* expression levels in RNAi and OX lines, quantified via RT-qPCR using *cons6* as the control. Statistical significance was evaluated by unpaired 2-tailed *t*-test; error bars indicate Se (*P* < 0.05 considered significant). **D)** Hairy root phenotypic analysis comparing EV, RNAi, and OX lines, accompanied by nodule structural assessment using *USDA110::GUS* staining to visualize bacteroid. Whole-mount images of nodulated roots and corresponding cross-sections confirm spatial expression patterns. Scale bars: 0.2 mm (A, top), 1 mm (A, bottom), 5 mm (D, top), and 2 mm (D, bottom).

One of the bHLH TFs, encoded by *Glyma.03G105700*, a Myc-like *GmbHLH121*, was identified as a strong candidate for nodulation regulation. This identification was based on its enrichment in the phloem at 72 hpi and 21 dpi. The high expression pattern observed in root hair-stripped samples following rhizobial inoculation further supports its role in nodulation (Supplementary Fig. S4). Promoter activity analysis using GUS reporter assays demonstrated phloem-specific induction of the *GmbHLH121* promoter in response to rhizobial infection (Fig. 7A). This finding was consistent with phloem-specific responses observed in TRAP-seq data. To functionally characterize *GmbHLH121*, hairy root transformation was performed to generate transgenic lines with either RNAi-mediated knockdown (RNAi) or overexpression (OX). Phenotypic analysis revealed that RNAi lines exhibited increased nodule numbers, while OX lines did not differ in nodulation, demonstrating the TF's critical role in phloem-specific nodule formation (Fig. 7B). Real-time PCR confirmed successful genetic modifications, with decreased *GmbHLH121* expression in RNAi lines and increased expression in OX lines (Fig. 7C).

To assess the specificity of the RNAi construct, we first performed phylogenetic analysis and sequence alignment of *GmbHLH121* and its closest homologs (Supplementary Fig. S5, A and B). These analyses revealed high sequence similarity in the targeted region, with *GmbHLH122* showing 95% identity and *GmbHLH123* showing 89% identity. RT-qPCR analysis (Supplementary Fig. S5C) further demonstrated that while *GmbHLH122* was co-silenced in RNAi lines—likely due to its high sequence similarity—*GmbHLH123* expression was not significantly affected (*P* = 0.936). These results support the conclusion that the RNAi construct specifically and effectively targets *GmbHLH121* and its closest homolog *GmbHLH122*, without inducing broader off-target effects.

Histological analysis indicated that nodule structures remained consistent across all transgenic lines, suggesting that *GmbHLH121* primarily regulates nodule number rather than morphology (Fig. 7D). Interestingly, the expression of early-stage nodulation markers, such as *ENOD40*, *NIN*, and *NSP*, did not differ among transgenic plants (Supplementary Fig. S5D). This suggests that the pathway controlling nodule numbers might operate independently of these genes or function downstream.

## Discussion

### Establishment and functional validation of a phloem-specific TRAP-seq system

The establishment of a phloem-specific TRAP-seq system represents a significant advancement in elucidating translational regulatory mechanisms in the phloem during nodulation. By utilizing the *Glyma.01G040700* promoter, we successfully targeted ribosome-associated mRNAs in phloem tissues. However, initial assessments revealed that the endogenous activity of this promoter alone was insufficient to achieve optimal ribosome-tagging efficiency for TRAP-seq. To address this limitation, we introduced the GAL4-UAS system, which significantly enhanced expression levels while maintaining phloem specificity.

The successful implementation of the GAL4-UAS amplification strategy underscores the necessity of optimizing promoter-driven expression in tissue-specific TRAP-seq applications. While our previous study effectively utilized endogenous promoters for TRAP-seq in cortex tissue (Song et al. 2022), our findings suggest that additional regulatory elements may be required for applications targeting the phloem in soybean. One of the key advantages of this optimized system is its ability to capture dynamic translational changes in the phloem during nodulation. Time-course TRAP-seq experiments provided high-resolution insights into phloem translatome reprogramming, facilitating the identification of TFs and signaling molecules crucial for phloem regulation. Furthermore, this system minimizes contamination from adjacent cell types, thereby increasing the reliability of detected gene expression changes and enhancing the accuracy of subsequent functional analyses. Despite these advances, several limitations must be considered. The translational changes observed in the phloem do not necessarily indicate phloem-specific regulation, as similar changes may occur in neighboring root tissues. A more comprehensive comparative approach incorporating TRAP-seq datasets from multiple root tissues is required to rigorously delineate phloem-specific translational regulation.

While our previous research included similar experiments in cortex tissues, data from additional root tissues remain insufficient. To improve the specificity of phloem regulation, integration with scRNA-seq analysis is essential. Although scRNA-seq data for nodules are available (Liu et al. 2023; Cervantes-Pérez et al. 2024), root-specific scRNA-seq data comparing pre- and post-rhizobial infection states are still lacking. This data gap constrains our ability to precisely map phloem-specific expression changes. Future studies should prioritize generating these datasets and developing computational strategies to enhance the resolution of tissue-specific gene expression analysis. Nonetheless, in the absence of such datasets, our TRAP-seq analysis provides a crucial framework for understanding the functional changes associated with nodulation specifically within the phloem. By offering insights into phloem-specific translational regulation, our study establishes a foundational platform that can be leveraged for future investigations into legume symbiosis and phloem-mediated regulatory networks.

### Phloem-driven immune regulation in legume-Rhizobium interactions

Rhizobium infection initiates in root hairs and spreads to the cortex, during which the *Nod factor* plays a crucial role in modulating innate immune signaling through interactions with the host's *pattern recognition receptor*s (*PRR*s) to facilitate symbiosis. Generally, *PRR*s recognize *microbial-associated molecular patterns* to activate defense responses, but the Nod factor secreted by Rhizobium partially suppresses *PRR* signaling, thereby modulating the host immune response and enabling discrimination between pathogenic and symbiotic microbes (Liang et al. 2013; Cao et al. 2017). Following initial infection in root hairs, as it progresses into the cortex, the immune response remains finely regulated to facilitate Rhizobium establishment, with genes such as *RIN4*, *WRKY22*, and *CDPK*s involved in sophisticated mechanisms that distinguish pathogens from Rhizobium (Yu et al. 2018; Feng et al. 2021; Wang et al. 2022a; Tóth et al. 2025). This regulatory mechanism is essential for maintaining the balance between symbiosis and immunity.

CSs generally function as selective barriers within endodermal cells, regulating water and nutrient uptake while restricting pathogen invasion (Robbins et al. 2014). While apoplastic pathway-based pathogen infection is effectively blocked by CSs, the potential for infection through the symplastic pathway remains primarily facilitated by PD. Our study provides strong evidence that a sustained increase in the expression of various cell wall-modifying enzymes in the phloem from early to late infection stages strongly correlates with PD regulation, suggesting that Rhizobium infection may induce structural modifications that enhance PD permeability, thereby facilitating increased molecular transport, particularly macromolecule movement, between the phloem and nodule. Previous studies reported increased macromolecule transport from the phloem to nodule primordia following Rhizobium infection (Complainville et al. 2003), further supporting the hypothesis that PD opening between the phloem and cortex is a key regulatory mechanism in both infection and symbiosis. Recent findings (Shen et al. 2025) further underscore the importance of apoplastic barriers such as CSs in nodulation, showing that MYB36-dependent CS formation is essential not only for maintaining symplastic connectivity but also for effective root-to-shoot signaling and nodule development in *L. japonicus*. This highlights a broader role for structural barriers in coordinating long-distance communication and local symbiotic responses.

In our study, we confirmed a significant increase in the expression of *GmBAK1* (*Glyma.05G119600*), *GmWRKY22* (*Glyma.16G031900*), *GmLRRs* (*Glyma.09G210600*, *Glyma.01G010700*, *Glyma.08G128800*, *Glyma.18G093900*), and *GmCDPKs* (*Glyma.10G084000*, *Glyma.02G192700*) in the phloem at 72 hpi, supporting the hypothesis that the phloem serves as a key immune-regulatory hub to control infection spread. Although we did not conduct direct functional validation assays, this transcriptomic evidence suggests that the phloem transiently participates in immune signaling during the early phase of nodulation. By 21 dpi, the expression of most of these genes—excluding *GmCDPKs*—was either significantly downregulated or unchanged relative to mock controls, indicating that the phloem-associated immune response is largely transient and gene-specific. Unlike the fine-tuned immune gene regulation observed in the cortex and root hairs, the phloem displayed a more pronounced upregulation of multiple immune-related genes during early infection. This pattern suggests that beyond immune modulation, the phloem may play a proactive role in restricting pathogen spread. Furthermore, we propose that the accumulation of immune-regulatory proteins in the phloem may contribute not only to local pathogen resistance but also to symbiotic signaling, potentially coordinating immune cues from the phloem to the cortex.

Thus, our findings suggest that the phloem functions not only as a transport conduit but also as a key regulatory tissue that precisely modulates infection spread through immune responses and molecular exchange during Rhizobium infection. Further studies are necessary to explore the interplay between PD regulation and immune networks within the phloem at a more mechanistic level.

### Regulation of AAP2 and AAP8 expression and their role in nitrogen fixation in root nodules

In legume root nodules, bacteroids fix atmospheric nitrogen (N_2_), converting it into ammonia (NH_3_), which is then transported into host cells via the Nod26 channel and subsequently assimilated into glutamine and asparagine (Fortin et al. 1987). The transport of fixed nitrogen varies across species; temperate legumes (e.g. pea and alfalfa) primarily utilize amide forms, whereas tropical legumes transport nitrogen as ureides, such as allantoin and allantoic acid (Miao et al. 1991; Tajima et al. 2004). These organic nitrogen compounds are exported from nodules into the xylem and distributed throughout the plant via interactions with the phloem (Herridge et al. 1978).

The endodermis of root nodules restricts apoplastic nitrogen transport, necessitating symplastic movement. In legumes, UPS1 transporters, such as *PvUPS1* in French bean and *GmUPS1-1/1-2* in soybean, play crucial roles in transporting allantoin and allantoic acid (Pélissier et al. 2004; Collier and Tegeder 2012). Meanwhile, in Arabidopsis, AAP2 and AAP6 mediate amino acid exchange between the xylem and phloem, facilitating efficient nitrogen redistribution (Hunt et al. 2010; Zhang et al. 2010). AAP8 also functions as a key regulator of source-to-sink nitrogen partitioning, influencing phloem nitrogen transport and seed development (Santiago and Tegeder 2016). Additionally, in legumes, AAP and UPS1 transporters are localized to xylem parenchyma and transport phloem, suggesting their role in transient nitrogen storage and organic nitrogen transfer (Pélissier et al. 2004; Tegeder et al. 2007).

Our studies identified enrichment of the GO term “regulation of amino acid transport” at 72 hpi and 21 dpi (Fig. 3, A and C). A detailed examination of *AAP* gene expression in phloem revealed that *GmAAP2* (*Glyma.11G107000*, *Glyma.06G156700*) expression is significantly upregulated at 72 hpi in phloem. Given that AAP2 is a major transporter responsible for transferring amino acids from the xylem to the phloem, this may play a role in providing amino acids and energy sources required for cell proliferation and expansion during early nodule formation. However, by 21 dpi, *GmAAP2* (*Glyma.11G107000*, *Glyma.12G032000*) expression showed a declining trend, likely due to reduced dependency on external amino acid supply as nodules mature. This decrease may also be attributed to the ability of bacteroids to autonomously fix nitrogen, reducing the necessity for phloem-mediated nitrogen input. These findings suggest that *AAP2* plays a pivotal role in regulating early nitrogen supply, while its function gradually diminishes as nodules mature.

AAP8 is a major regulator of amino acid loading into the phloem, potentially facilitating the efficient distribution of nitrogen fixation products to the shoot and seeds in Arabidopsis. Our findings indicate that *GmAAP8* expression remained unchanged at 72 hpi but significantly increased at 21 dpi. This suggests that as nodules mature and nitrogen fixation becomes active, the demand for redistribution of glutamine, asparagine, and ureides (allantoin, allantoic acid) to sink tissues (e.g. seeds and emerging leaves) increases. Therefore, the elevated expression of *GmAAP8* in the phloem could potentially serve as a key regulatory mechanism for the efficient partitioning of fixed nitrogen.

Our study suggests that in soybean, *GmAAP2* is primarily responsible for early nitrogen supply, whereas *GmAAP8* may contribute to the later-stage distribution of nitrogen fixation products. *GmAAP2* may be involved in nitrogen acquisition and supports nodule growth during the early stages, whereas *GmAAP8* enhances nitrogen allocation to developing tissues at later stages, ultimately optimizing nitrogen use efficiency. Future research should focus on elucidating the regulatory networks governing their expression and integrating these insights into crop improvement strategies for enhanced nitrogen utilization efficiency.

### Regulation of phloem-associated *PME* and *PMEI* in nodulation: insights into cell wall modification and symplastic transport

Nodulation in legumes is a complex developmental process that appears to require intricate coordination of symbiotic signaling, cell wall remodeling, and PD regulation. In this study, our findings suggest that the majority of *PME* genes activated at 72 hpi and 21 dpi contain a *PMEI* domain. Previous research suggests that *PME* isoforms possessing an intrinsic *PMEI* domain may have a more refined and responsive regulatory mechanism compared to those lacking it (Bosch and Hepler 2005). This regulation appears to be influenced by pH, as interactions between PME and PMEI are pH-dependent, leading to dynamic shifts in enzymatic activity (Hocq et al. 2024). Additionally, PMEIs play a multifaceted role beyond direct inhibition, contributing to broader regulatory networks involved in cell wall remodeling and stress responses (Wormit and Usadel 2018). This inherent regulatory capacity could be particularly advantageous in highly dynamic processes such as rhizobial infection and nodulation, where timely modifications of the cell wall might be essential for both bacterial entry and nodule organogenesis.

Cell wall modifications mediated by *PME* and *PMEI* seem to play a role in the spatial and temporal regulation of nodulation. During rhizobial infection, localized cell wall acidification and reactive oxygen species (ROS) signaling in root hair tips were reported to contribute to symbiotic establishment (Ramu et al. 2002). The activation of ROS signaling during infection may lead to pH fluctuations that influence *PME* activity, potentially promoting rapid and dynamic modifications of the cell wall at the site of bacterial entry. Since *PMEI* binding affinity to *PME* is pH-dependent, transient acidification of the infection site could weaken *PMEI* inhibition, allowing for localized *PME* activation and subsequent remodeling of the cell wall. This mechanism may facilitate the controlled relaxation of cell wall structures necessary for bacterial invasion, infection thread formation, and the early stages of nodule development.

In addition to their potential role in early infection, *PME* and *PMEI* gene regulation may contribute to the long-distance coordination of nodulation. Our TRAP-seq analysis revealed elevated expression of several *PME–PMEI* genes in phloem-associated tissues, suggesting their involvement in modulating PD permeability. Such modulation could influence the systemic movement of signaling molecules critical for nodule development. Supporting this idea, studies in *M. truncatula* demonstrated that callose degradation enhances PD conductivity, thereby promoting rhizobial infection and increasing nodulation efficiency (Gaudioso-Pedraza et al. 2018). Given these functional parallels in PD regulation, it is plausible that *PME–PMEI* activity in the phloem fine-tunes symplastic connectivity, facilitating long-distance signaling and resource allocation necessary for efficient nodulation.

To functionally validate this hypothesis, we examined the role of *GmPME25*, a phloem-expressed *PME* gene identified in our dataset. A *GmPME25p:DsRED* reporter confirmed promoter activation in the root phloem at 72 hpi, indicating involvement during early infection stages (Fig. 4F). Phloem-specific overexpression of the *PME* domain significantly increased nodule number and reduced callose deposition, whereas overexpression of the *PMEI* domain resulted in enhanced callose accumulation and a concomitant decrease in nodule formation (Fig. 5, B and D). These opposing phenotypes support the idea that *PME25*-mediated modulation of PD permeability influences nodulation efficiency by regulating symplastic signaling. In contrast, RNAi-mediated suppression of *PME25* did not significantly alter either trait, likely due to genetic redundancy among the *PME* family members.

Further regulatory complexity arises from environmental and hormonal signals that influence *PME* and *PMEI* expression. *Cis*-element analysis of their promoter regions identified motifs responsive to jasmonic acid (MeJA), abscisic acid (ABA), and hypoxia-inducible elements. These findings suggest that *PME* and *PMEI* genes are tightly regulated by dynamic cues encountered during nodulation. Given the roles of MeJA and ABA in defense and abiotic stress responses, their regulation of *PME–PMEI* activity may help balance symbiotic signaling with overall plant fitness. However, the downstream mechanisms linking these hormonal and environmental signals to specific cell wall remodeling events remain to be elucidated.

Taken together, these findings suggest that PME and PMEI function as multifaceted regulators in soybean nodulation. Beyond their canonical role in cell wall remodeling, these enzymes contribute to infection site plasticity, long-distance symbiotic signaling, and environmental adaptation during nodule development. Nonetheless, further research is required to clarify the precise molecular mechanisms by which PME and PMEI coordinate these processes, particularly in relation to hormonal crosstalk and systemic signaling.

### The phloem as a site of transcriptional control in nodulation

The regulatory dynamics of TFs within the phloem play a central role in the legume-rhizobium symbiotic interaction. This study refines previous transcriptional models by providing high-resolution insights into phloem-specific regulatory mechanisms, reinforcing its function as an active signaling hub rather than a passive transport conduit. Prior research established that *MYB* governs flavonoid biosynthesis to enhance rhizobial infection (Liu et al. 2013), *bHLH* modulates nodule vascular patterning and development in *M. truncatula* (Godiard et al. 2011), *C2H2* and *NAC* mediate symbiotic signaling and defense responses (De Zélicourt et al. 2012; Liu et al. 2022), and *WRKY* regulates pathogen defense and metabolic activation (Yang et al. 2025). Our findings confirm that these TFs undergo significant expression shifts within the phloem, underscoring their role as a regulatory nexus in nodulation. Our TRAP-seq data support this possibility, revealing distinct expression dynamics of key TFs within the phloem during early nodulation. In the early phase, *ERF* plays a central role in ethylene signaling, while *C2H2* and *NAC* contribute to early stress response and nodule development. *MYB* supports root hair infection and flavonoid biosynthesis, *WRKY* is involved in metabolic defense regulation, and *bHLH* influences root architecture and rhizobial attachment. Notably, we highlight the dynamic expression of *ERF* and *ARF* as key mediators of phloem-driven long-distance signaling during nodulation.

*ERF* exhibited the highest upregulation during the early nodulation stage (72 hpi), suggesting that ethylene signaling through the phloem is integral to nodulation initiation. Previous studies established that *ERF* facilitates infection thread formation and orchestrates nodulation onset within the root (Kawaharada et al. 2017), our phloem-targeted TRAP-seq data revealed significant enrichment of *ERF* transcripts in phloem tissues during early rhizobial infection. However, we did not detect phloem-specific upregulation of ethylene biosynthesis genes (e.g. 1-aminocyclopropane-1-carboxylic acid synthase, 1-aminocyclopropane-1-carboxylic acid oxidase) or core signaling components, and since ethylene is a gaseous hormone that diffuses passively across membranes, its direct systemic transport through the phloem is unlikely. Notably, although the precursor ACC (1-aminocyclopropane-1-carboxylic acid) can be transported through vascular tissues and converted to ethylene in target organs, our dataset did not show strong evidence for ACC biosynthetic gene enrichment in phloem. Thus, our findings do not support the hypothesis of shoot-derived ethylene acting via phloem transport. Instead, phloem-localized *ERF* activity may reflect localized or alternative signaling cues, possibly involving other stress- or hormone-related pathways. Furthermore, since *ERF* genes are also known to be induced by a wide range of abiotic and biotic stresses, including UV-B (Wang et al. 2022c), cold (Stockinger et al. 1997), and pathogen stimuli (Oñate-Sánchez and Singh 2002), the observed *ERF* expression may be governed by complex regulatory networks beyond ethylene signaling alone.

Conversely, *ARF* was markedly downregulated during the late nodulation stage (21 dpi), suggesting that auxin signaling suppression through the phloem plays a crucial role in refining nodulation dynamics. Prior research has demonstrated that *miR167c*, expressed within vascular tissue, targets *GmARF6* and *GmARF8* to regulate nodule formation within the root (Yan et al. 2015; Zhang et al. 2021). In alignment with these findings, our data reveal a pronounced reduction in *ARF* expression, particularly *GmARF8* (*Glyma.02G239600*, *Glyma.11G204200*), within the phloem at 21 dpi. This suggests that *miR167c*-mediated suppression of *ARFs* may extend beyond local root regulation, instead constituting a shoot-to-root long-distance signaling mechanism via the phloem. The downregulation of auxin signaling at this stage likely promotes nodule maturation while preventing excessive nodule formation, optimizing symbiotic efficiency.

Taking together, this study presents a refined model of nodulation regulation by emphasizing the role of phloem-specific transcriptional changes. The early upregulation of *ERF* genes in the phloem may reflect stress- or hormone-responsive signaling during nodulation initiation. In contrast, the late downregulation of *ARF* genes suggests that auxin signaling is modulated in the phloem to support nodule maturation and restrict excessive formation. By integrating high-resolution transcriptional profiling, we provide a deeper understanding of the spatial and temporal dynamics of nodulation control and highlight the phloem's role in coordinating regulatory signals during the legume-rhizobium symbiosis.

### Phloem-localized *GmbHLH121* and its role in nodule development

*GmbHLH121*, one of the 355 homologous bHLH TFs identified in *G. max*, is classified within subclade IVa, the most expansive among the 25 established bHLH subclades. Notably, legume species exhibit a greater diversification within this subclade than other plant families, suggesting a lineage-specific expansion and functional divergence (Brown and Hudson 2015; Suzuki et al. 2021). Within subclade IVa, *GmbHLH121* is assigned to Subgroup 1, which is characterized by a broader range of expression profiles, in contrast to Subgroups 2 and 3 that predominantly exhibit nodule- and root-specific expression patterns.

Recent findings established *GmbHLH300* as a negative regulator of nodulation and nitrogen fixation, particularly under iron-deficient and iron-excess conditions, thereby playing a pivotal role in iron homeostasis within nodules (Wu et al. 2023). Mechanistically, *GmbHLH300* directly associates with the promoter regions of *ENOD93*, a positive regulator of nodulation, and *GmLba*, a gene encoding a key enzyme for leghemoglobin biosynthesis, thereby modulating their transcriptional activity.

In our investigation, *GmbHLH121* emerged as a previously uncharacterized regulatory factor in nodulation, exhibiting a significant role in the response to rhizobial inoculation and modulating nodule numbers. However, differential gene expression analyses revealed that key early nodulation marker genes exhibited no substantial variation in expression levels across EV, overexpression, and knockdown lines. This observation suggests that *GmbHLH121* does not act as a direct transcriptional regulator of these early nodulation-associated genes, indicating a more intricate regulatory mechanism that warrants further in-depth functional characterization.

## Materials and methods

### Plant materials, growth conditions, and bacterial strains

Soybean (*G. max*) cultivar Williams 82 was used for all experiments. Seeds were surface sterilized and germinated on sterilized germination paper or in a 3:1 mixture of vermiculite and perlite under controlled conditions (16 h light/8 h dark, 26 °C/23 °C light/dark, 80% relative humidity). Seedlings were inoculated with *Bradyrhizobium diazoefficiens* strain USDA110(WT) or USDA110(GUS), grown at 30 °C in HM medium (Cole and Elkan 1973) supplemented with appropriate antibiotics for 3 d. After incubation, bacterial cultures were centrifuged at 5,000 × *g* for 10 min, washed twice with sterile DI water, and resuspended to an OD_600_ of 0.1 in sterile DI water for inoculation. Plants were grown in nitrogen-free B&D medium (Broughton and Dilworth 1971) or supplemented with 0.5 mm NH_4_NO_3_ for noninfected controls. Root samples were harvested at designated time points in a sequential manner, ensuring that nodules were carefully removed prior to collection. The harvested roots were immediately frozen in liquid nitrogen and stored at −80 °C for further analysis. All experiments included 3 biological replicates.

### Cloning and plasmid construction

Promoter sequences of *Glyma.01G040700* and target genes were amplified from Williams 82 genomic DNA. The promoter, *GAL4-VP16*, *nos* terminator, and *5xUAS* were cloned into *pSoyGUS* (Hossain et al. 2020) (KpnI, PstI digested) using Gibson assembly. Likewise, the promoter fragment, *GAL4-VP16*, *nos* terminator, *5×UAS*, and HF (His-Flag)-GmRPL18 were assembled into the pCAMGFP–CvMV–GWi binary vector (Graham et al. 2007) (KpnI, PstI digested) using Gibson assembly. The *GAL4-VP16* and *5×UAS* sequences were PCR-amplified from the “Haseloff” lines (Haseloff 1998). All primers used for cloning are listed in Supplementary Table S8.

For Ox constructs, *pCAM-Ruby-Ox* plasmid was generated by linearizing *pSoyGUS* with PstI and SacII, followed by Gibson Assembly with PCR-amplified fragments of the Ruby gene from the *35S:RUBY* plasmid (He et al. 2020), and the *Ox* promoter from a corresponding template. The coding sequence of *GmbHLH121* (*Glyma.03G105700*) was amplified from cDNA templates and cloned into PstI site by Gibson assembly.

For RNAi constructs, *pCAMRUBY-CvMV-GWi* binary vector was established by cloning the gateway RNA interference (GWi) sequences into PstI site of *pCAM-Ruby-Ox* vector. Approximately 160 bp gene-specific fragment of *GmbHLH121* was amplified using cDNA and cloned into *pDONR/Zeo*, followed by recombination into the *pCAMRUBY-CvMV-GWi* binary vector. For the Promoter::GUS construct, ∼2.0 kb sequences of *GmbHLH121* promotor were amplified from soybean gDNA and cloned by Gibson assembly into the *pSoyGUS* vector (KpnI, PacI digested).

To functionally dissect PME25 domains, 2 truncated versions were generated under the control of the phloem-specific promoter (*Glyma.01G040700*). The *pCAMGFP_ProPH_PME25_truncated(SP-TM-PME)* construct was assembled using a 2.6 kb phloem promoter fragment amplified from genomic DNA, a 75 bp signal peptide and transmembrane domain (SP-TM) region, and a 930 bp PME or a 444 bp PMEI domain amplified from cDNA. All fragments were assembled into the KpnI/PstI-digested pCAMGFP/CvMV-GWi binary vector via Gibson assembly.

For gene silencing of *GmPME25*, an RNAi construct (*RNAi-GmPME25*) was generated using the Gateway-compatible pCAMGFP-gw-RNAi binary vector. A gene-specific fragment was first cloned into *pDONR/Zeo* and then recombined into the binary RNAi vector via LR recombination.

### Soybean hairy root transformation and microscopy

*Agrobacterium rhizogenes* strain K599 carrying target constructs was used for hairy root transformation as described previously (Song et al. 2021). Transgenic roots were selected based on GFP fluorescence using a dissecting fluorescence microscope or Ruby color by visual inspection before and after inoculation with *B. diazoefficiens*. Nodule numbers were recorded 4 weeks postinoculation. Harvested roots were flash-frozen in liquid nitrogen for molecular analysis. For histological studies, nodules were hand-sectioned and subjected to GUS staining before visualization. Stained sections were examined using bright-field microscopy (Leica DM5500B). Statistical analysis included at least 10 plants per replicate, and Student's *t*-test or ANOVA followed by Tukey's HSD test were used for significance testing.

### TRAP and RNA sequencing (TRAP-seq)

TRAP was performed using His-FLAG-tagged GmRPL18 as described by Zanetti et al. (2005) with modifications (Song et al. 2022). Phloem-enriched hairy root tissues expressing HF-GmRPL18 were harvested at 72 hpi and 21 dpi. Approximately 10 g of root material per sample was used for immunoprecipitation, yielding at least 400 ng of mRNA for library construction. Three biological replicates were analyzed per time point.

### RNA-seq library preparation and sequencing

Total RNA extracted from TRAP samples was shipped on dry ice to the University of Missouri Genomics Technology Core. Libraries were prepared using the TruSeq Stranded mRNA Kit (Illumina) and sequenced (75 bp paired-end) on an Illumina NextSeq 500 platform.

### Bioinformatics analysis of TRAP-seq data

FASTQ files were quality-checked using MultiQC (version 1.9) (Ewels et al. 2016), and low-quality reads were filtered. High-quality reads were mapped to the *G. max Wm82.a4.v1* genome using STAR (version 2.7.4a) (Dobin et al. 2013), and a raw expression matrix was generated using HTSeq (version 0.14) (Anders et al. 2015). Differential expression analysis was performed with edgeR (version 3.32.1) (Robinson et al. 2010). Heatmaps were generated using log_2_–fold change values for significant genes. The identified DEGs were analyzed and annotated using PlantTFDB v5.0 (Tian et al. 2019) to identify TFs within the DEGs. RNA-seq data were deposited into the NCBI GEO database (GSE292049).

### Functional and pathway enrichment analysis

GO enrichment analysis was performed using the tool described by Morales et al. (2013) and results were further summarized using REVIGO (Supek et al. 2011) to remove redundant GO terms. KEGG pathway analysis was conducted using g:Profiler (Raudvere et al. 2019). All analyses were conducted using default parameters unless otherwise specified.

### *Cis*-regulatory element analysis

*Cis*-element analysis was conducted on 2 kb promoter sequences using PlantCARE (Lescot 2002). Promoter sequences were submitted to the PlantCARE database to identify regulatory motifs. The analysis included scanning for hormone-responsive elements, stress-related regulatory sites, and TF binding motifs. All parameters were set to default unless otherwise specified.

### Histochemical GUS staining

GUS staining was conducted following Jefferson et al. (1987). Transgenic roots were incubated overnight at 37 °C in GUS staining solution (1 mg/mL X-Gluc, 100 mm sodium phosphate buffer, pH 7.0, 1 mm EDTA, 0.05% Triton X-100, 5 mm potassium ferrocyanide, 5 mm potassium ferricyanide). Stained roots were imaged using a Leica DM5500B microscope.

### Callose staining of soybean roots

Callose deposition was visualized in hand-sectioned soybean roots using an optimized aniline blue staining protocol. Root segments were fixed in Navashin's fixative (70% ethanol, 20% formaldehyde, 10% glacial acetic acid) for 2 h at room temperature and rinsed 3 times in distilled water. Fixed samples were sectioned (50–100 *μ*m) using a razor blade and stained in 0.1% (w/v) aniline blue prepared in 0.5 m potassium phosphate buffer (pH 8.0) for 10 min at room temperature. Sections were briefly rinsed with same buffer and imaged immediately under a fluorescence microscope (Zeiss Axiovert 200M and Nikon Eclipse) equipped with UV filter sets.

### Real-time-qPCR

Total RNA was extracted from transgenic roots and treated with DNase I. cDNA was synthesized from 2 *μ*g of total RNA using M-MLV Reverse Transcriptase (Promega). RT-qPCR was performed using SYBR Green PCR mix (ABI) on a Bio-Rad CFX96 system. Relative transcript levels were normalized against the Cons6 reference gene (Libault et al. 2008). Primer sequences are listed in Supplementary Table S8.

### Immunoprecipitation and western blot analysis

Total proteins were extracted from ∼200 mg of transgenic root tissues. For immunoprecipitation, 1 mg of protein extract was incubated with anti-FLAG M2 Affinity Gel (Sigma) following the manufacturer's protocol. Western blotting was performed using monoclonal anti-FLAG-HRP antibody (1:3,000, Sigma). Detection was carried out using enhanced chemiluminescence.

### Statistical analysis

Statistical analyses were performed using GraphPad Prism (version 8). For all quantitative experiments, the number of biological and technical replicates is indicated in the corresponding figure legends. RT-qPCR data were analyzed using the 2^−ΔΔCt^ method. Statistical significance was assessed using 2-tailed Student's *t*-test for comparisons between 2 groups, and 1-way ANOVA followed by Tukey's HSD post hoc test for multiple group comparisons. A *P*-value < 0.05 was considered statistically significant unless otherwise noted. Error bars in all graphs represent the standard error of the mean.

### Accession numbers

Sequence data from this article can be found in the GenBank/EMBL data libraries under accession numbers _.Ph (*Glyma.01G040700*), *GmPME25* (*Glyma.03G029100*), *GmARFs* (*Glyma.09G072200*, *Glyma.07G134800*, *Glyma.07G130400*, *Glyma.02G239600*, *Glyma.11G204200*), *GmbHLH121* (*Glyma.03G105700*), *GmbHLH122* (*Glyma.07G117600*), *GmBAK1* (*Glyma.05G119600*), *GmWRKY22* (*Glyma.16G031900*), *GmLRRs* (*Glyma.09G210600*, *Glyma.01G010700*, *Glyma.08G128800*, *Glyma.18G093900*), *GmCDPKs* (*Glyma.10G084000*, *Glyma.02G192700*), and *GmAAP2* (*Glyma.11G107000*, *Glyma.06G156700*, *Glyma.12G032000*).

## Supplementary Material

kiaf570_Supplementary_Data

## Data Availability

The RNA-seq data generated in this study have been deposited in the NCBI Gene Expression Omnibus (GEO), accession number GSE292049, and are publicly available.

## Supplementary data

The following materials are available in the online version of this article.

**Supplementary Figure S1.** Comparison of phloem-enriched gene sets identified by TRAP-seq and scRNA-seq analyses at early and late stages of nodulation.

**Supplementary Figure S2.** KEGG pathway enrichment analysis of DEGs in soybean root phloem.

**Supplementary Figure S3.** Callose deposition and PME25 expression in soybean root.

**Supplementary Figure S4.** Spatial expression profile of Glyma.03G132700 in soybean root phloem and other tissues.

**Supplementary Figure S5.** Homologous genes involved in RNA silencing.

**Supplementary Table S1.** DEGs identified by TRAP-seq in phloem tissue.

**Supplementary Table S2.** Venn diagram analysis of DEGs in soybean root phloem.

**Supplementary Table S3.** GO enrichment and KEGG pathway summary.

**Supplementary Table S4.** REVIGO GO summary.

**Supplementary Table S5.** List of PME and PMEI genes and their differential expression (fold change) identified by phloem TRAP-seq analysis.

**Supplementary Table S6.**  *Cis*-regulatory element analysis of PME and PMEI candidates identified by phloem TRAP-seq.

**Supplementary Table S7.** Transcription factor profiling in soybean root phloem: response to B.J. infection at 72 hpi and 21 dpi.

**Supplementary Table S8.** Primer list used for this paper.

## Dive Curated Terms

The following phenotypic, genotypic, and functional terms are of significance to the work described in this paper:

AAP8 Gramene: AT1G10010
AAP8 Araport: AT1G10010

AAP6 Gramene: AT5G49630
AAP6 Araport: AT5G49630

MIR167C Gramene: AT3G04765
MIR167C Araport: AT3G04765

RIN4 Gramene: AT3G25070
RIN4 Araport: AT3G25070

WRKY22 Gramene: AT4G01250
WRKY22 Araport: AT4G01250

NAC CHEBI: CHEBI:7421

AAP2 Gramene: AT5G09220
AAP2 Araport: AT5G09220

root AmiGo: PO:0009005

GUS Gramene: beta-glucuronidase
GUS Araport: beta-glucuronidase

GFP Gramene: green fluorescent protein
GFP Araport: green fluorescent protein
