# Supplementary material for: Phloem-specific translational regulation of soybean nodulation: Insights from a phloem-targeted TRAP-seq approach
Source: Plant Physiol. 2025 Nov 15;199(3):kiaf570. doi: 10.1093/plphys/kiaf570 (PMC12625658; doi:10.1093/plphys/kiaf570)
Supplement: kiaf570_Supplementary_Data [file kiaf570_supplementary_data.zip › Supplementary Data.pdf]

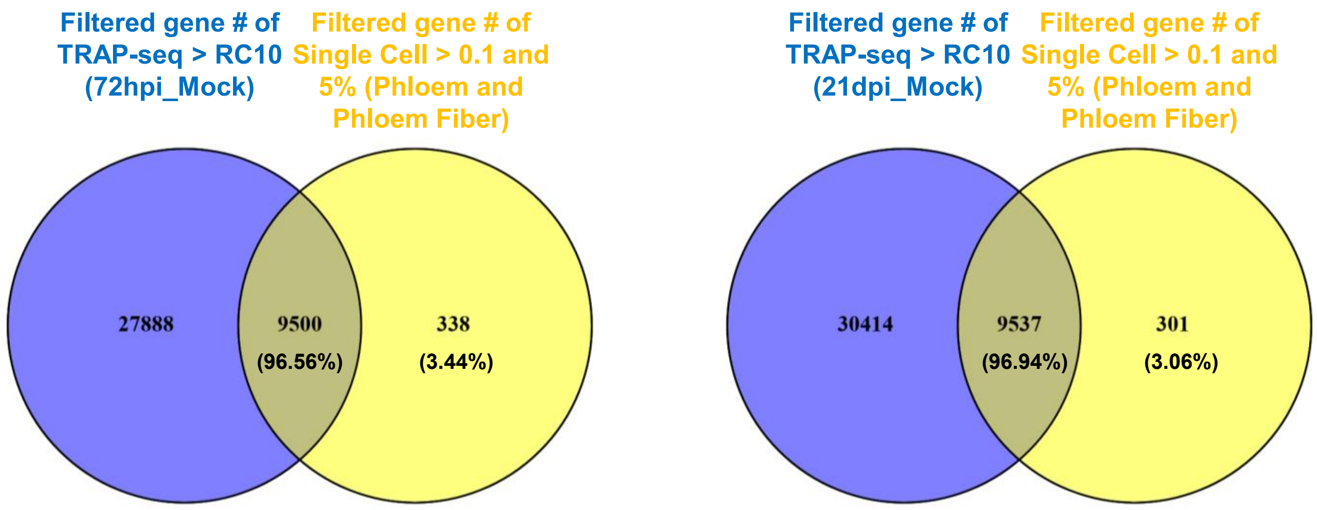

Supplementary Figure S1. Comparison of phloem-enriched gene sets identified by TRAP-seq and single-cell RNA-seq analyses at early and late stages of nodulation.

Venn diagrams illustrate the overlap between genes filtered from phloem TRAP-seq (RC10 > threshold) and those identified in single-cell RNA-seq data (expression > 0.1 and ≥5% of cells in phloem or phloem fiber clusters).

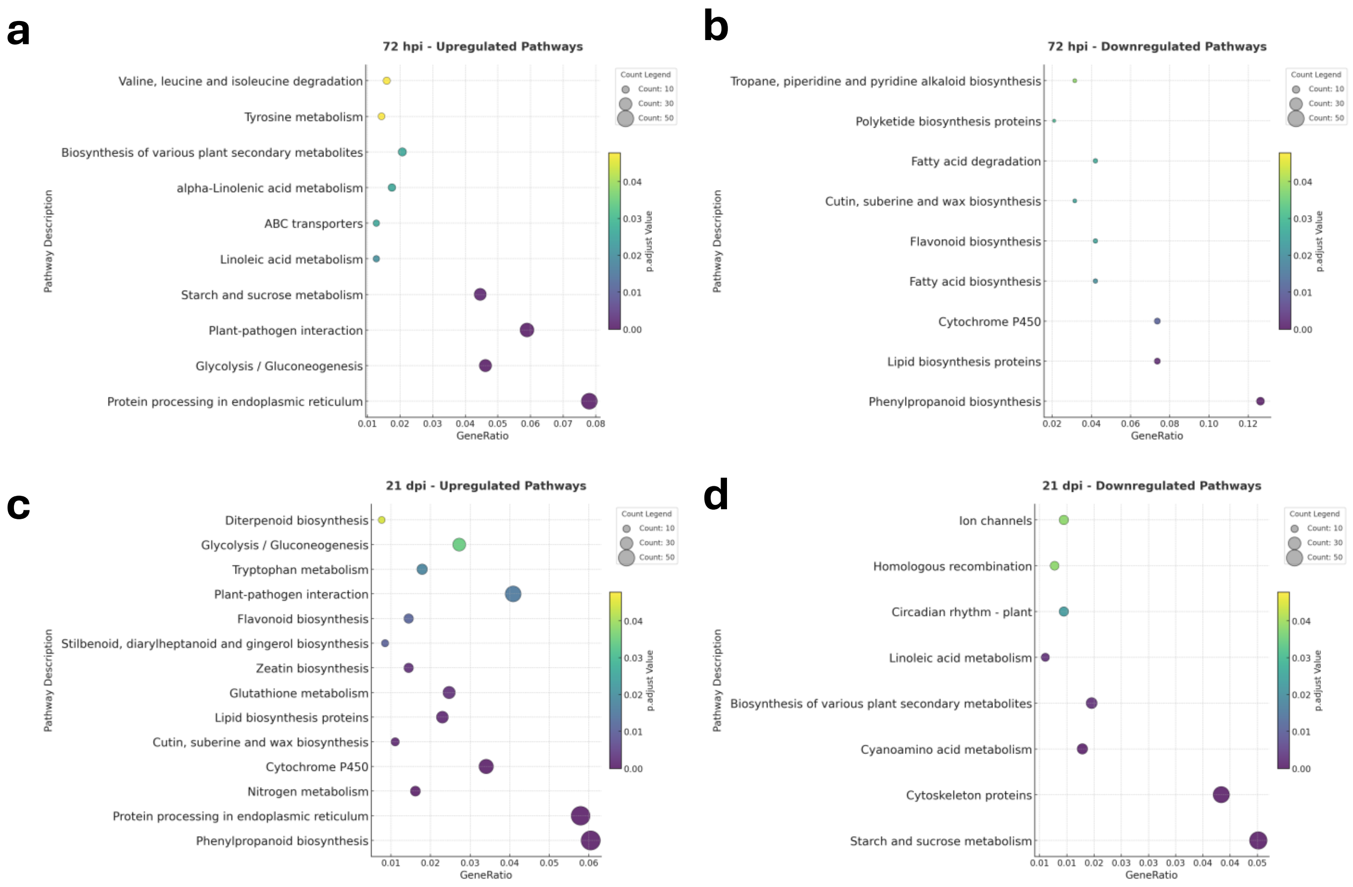

Supplementary Figure S2. KEGG Pathway Enrichment Analysis of Differentially Expressed Genes in Soybean Root Phloem.

KEGG pathway enrichment analysis of differentially expressed genes (DEGs) in soybean root phloem at 72 hpi and 21 dpi. Each bubble represents an enriched KEGG pathway, with bubble size corresponding to the number of DEGs mapped to the respective pathway and color intensity denoting statistical significance (-log10(P-value)). The x-axis represents the GeneRatio, defined as the proportion of DEGs associated with a given pathway relative to the total DEGs analyzed. Pathways are categorized based on expression patterns: (a) upregulated pathways at 72 hpi, (b) downregulated pathways at 72 hpi, (c) upregulated pathways at 21 dpi, and (d) downregulated pathways at 21 dpi. This analysis provides insights into dynamic transcriptional reprogramming within the root phloem in response to rhizobacterial symbiosis.

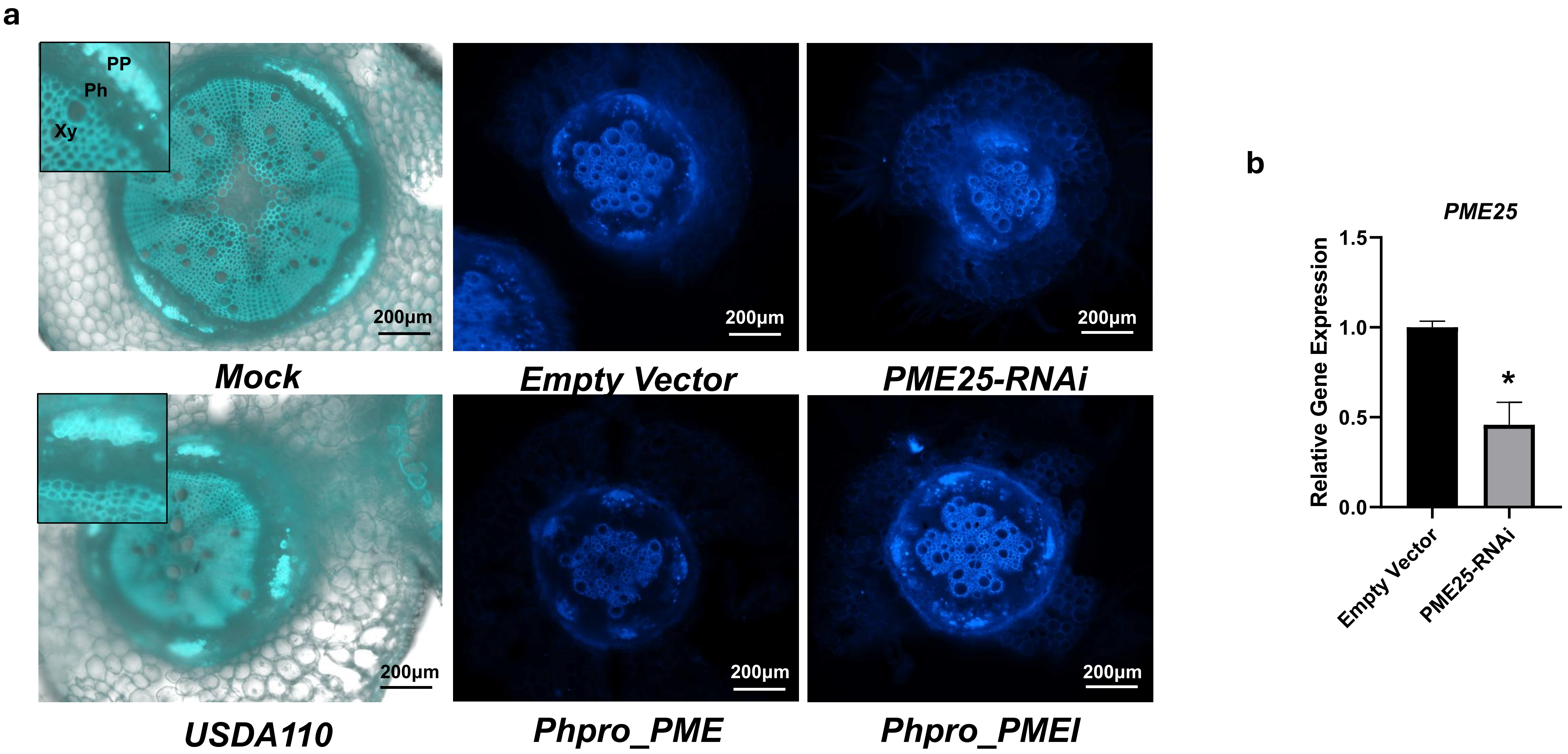

Supplementary Figure S3. Callose deposition and *PME25* expression in soybean root.

(a) Representative transverse sections of soybean roots stained with aniline blue to visualize callose accumulation (blue fluorescence). Brightfield images (left panels) are overlaid with fluorescence signals to show spatial distribution of callose. Insets highlight the phloem region (PP: protophloem; Ph: metaphloem; Xy: xylem).

Middle and right panels show corresponding fluorescence microscopy images acquired under UV illumination. Sections represent root samples from wild-type (Mock, USDA110) and transgenic lines (*PME25-RNAi*, *Phpro_PMEI*, *Phpro_PME*). Scale bars = 200 μm. (b) RT-qPCR analysis of *PME25* transcript levels in roots of transgenic *PME25-RNAi* lines compared to empty vector (EV) control. Expression of *Cons6* was used as an internal control. Statistical significance was evaluated by unpaired two-tailed t-test; error bars indicate SE ($p < 0.05$ considered significant, n=3).

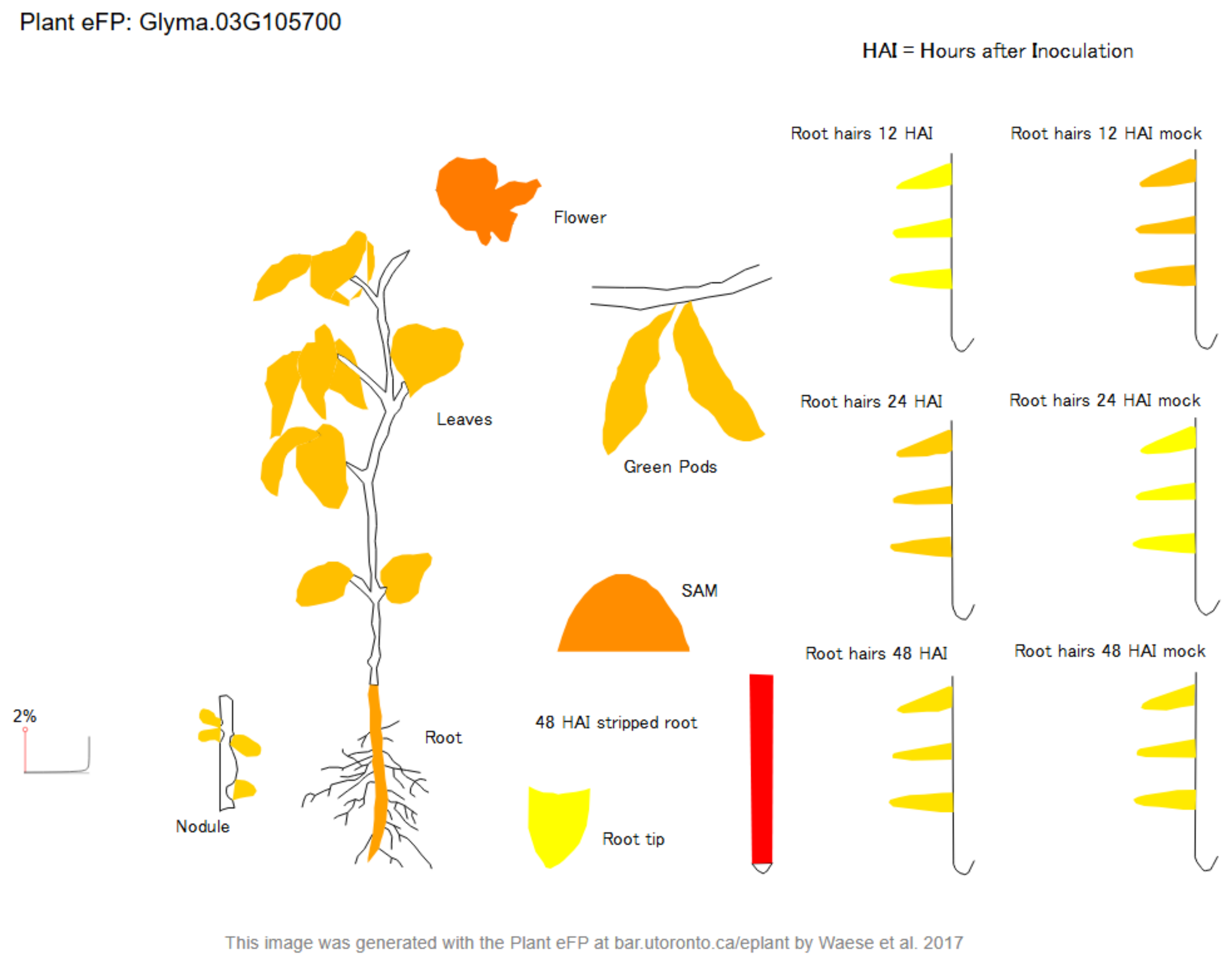

Supplementary Figure S4. Spatial Expression Profile of Glyma.03G132700 in Soybean Root Phloem and Other Tissues.

Expression distribution of *Glyma.03G132700* across various soybean tissues and developmental stages, visualized using the Plant eFP Browser. Gene expression levels are represented by a color gradient from yellow (low expression) to red (high expression), with grayscale indicating masked data (≥100% RSE, relative standard error).

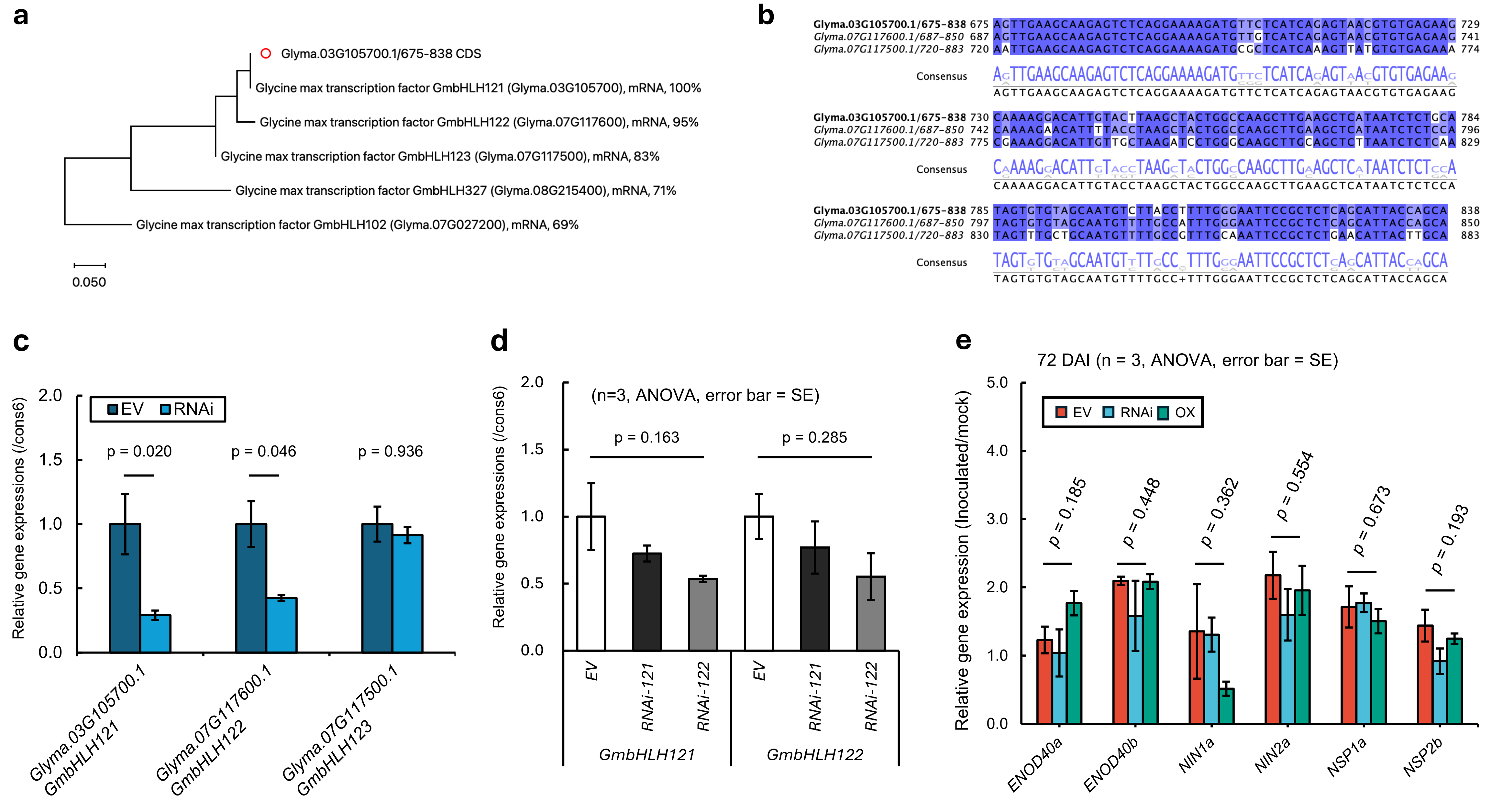

Figure S5. Homologous genes involved in RNA silencing.
(a) Phylogenetic tree generated by Neighbor Joining method using discontiguous MegaBLAST based on 164 bp RNAi fragment sequences. The scale bar represents the number of nucleotide substitutions per site, as calculated from BLAST pairwise distances. (b) Sequence alignment of the 164 bp RNAi target region among three soybean *GmbHLH* transcription factors. The entire sequence of *GmbHLH121* shares 95.1% identity with *GmbHLH122* and 83.5% identity with *GmbHLH123*. (c) RT-qPCR expression analysis of the three *GmbHLH* TFs. Error bars represent the standard error of the mean (n = 3). Statistical significance was determined using a Student's t-test. (d) RT-qPCR expression analysis of the duplicated *GmbHLH* TFs in each hairy root small RNAi transformation line. Error bars represent the standard error of the mean (n = 3). (e) RT-qPCR expression analysis of early nodulation-related genes were assessed in empty vector (EV), GmbHLH121 RNA interference (RNAi), and GmbHLH121 overexpression (OX) hairy roots using quantitative reverse transcription PCR (qRT-PCR). Gene expression was normalized against the reference gene *Cons6*, and fold changes were calculated relative to the EV control. Error bars represent standard error.
